# LoRa Technology in Flying Ad Hoc Networks: A Survey of Challenges and Open Issues

**DOI:** 10.3390/s23052403

**Published:** 2023-02-21

**Authors:** William David Paredes, Hemani Kaushal, Iman Vakilinia, Zornitza Prodanoff

**Affiliations:** 1 School of Engineering, College of Computing, Engineering and Construction, University of North Florida, Jacksonville, FL 32224, USA; 2 School of Computing, College of Computing, Engineering and Construction, University of North Florida, Jacksonville, FL 32224, USA

**Keywords:** Internet of Things (IoT), Flying Ad Hoc Networks (FANETs), Low Power Wide Area Networks (LPWANs), Wireless Sensor Networks (WSNs), mobility models, energy efficiency

## Abstract

The Internet of Things (IoT) and Flying Ad Hoc Networks (FANETs) have become hot topics among researchers because of the increased availability of Unmanned Aerial Vehicles (UAVs) and the electronic components required to control and connect them (e.g., microcontrollers, single board computers, and radios). LoRa is a wireless technology, intended for the IoT, that requires low power and provides long-range communications, which can be useful for ground and aerial applications. This paper explores the role that LoRa plays in FANET design by presenting a technical overview of both, and by performing a systematic literature review based on a breakdown of the communications, mobility and energy topics involved in a FANET implementation. Furthermore, open issues in protocol design are discussed, as well as other challenges associated with the use of LoRa in the deployment of FANETs.

## 1. Introduction

Due to what has been called the “wireless revolution” [1,2], the last couple of decades has seen an expansion in the number of connected devices aimed at making our lives easier at home and at work, as is the case with body sensors, light dimmers, vacuum cleaners, thermostats, refrigerators, autonomous vehicles, and many more items. Various sources claim that the number of connected devices has largely surpassed the number of connected people, establishing its potential magnitude in the order of tens of billions [3,4,5]. These connected devices need the means to communicate with each other as well as with the user, and this is frequently referred to as the Internet of Things (IoT). More formally, the IoT is the collection of objects that are equipped with sensors and actuators and are interconnected through a private or public network—generally the Internet in the latter scenario. The rise of the IoT, as is the case with many technological innovations, has been driven by the desire to improve quality of life in a cost-efficient manner [3].

While human communications often demand considerable bandwidth (e.g., on-demand video streaming and file sharing) and have little tolerance for delay (e.g., voice calls and videoconference), communications between objects have different characteristics, at least for the time being. IoT communications usually involve low data rates, where a relaxation of bandwidth constraints usually results in lower power consumption and a longer communication range, considering that these devices might be placed at isolated locations with no access to a power grid or Wide Area Networks (WANs) such as those available through internet service providers, mobile carriers, or proprietary deployments.

In a natural step in the development of wireless technologies, ad hoc networks were conceived as a way of connecting nodes when no centralized infrastructure is available, and the concept of mobility was immediately tied to this type of network [6]. Mobile Ad Hoc Networks (MANETs) have been used for military applications for many years [7]. As they increasingly connect more and more objects across the globe, MANETs have become one of the fundamental network paradigms in IoT. Moreover, the recent surge in the availability of consumer drones (also known as Unmanned Aerial Vehicles, or UAVs; terms used interchangeably hereafter), has given rise to the concept of Flying Ad Hoc Networks (FANETs).

### 1.1. Motivation

While existing wireless technologies—such as cellular and Wi-Fi—have been adapted for the IoT [8,9], Low Power Wide Area Networks (LPWANs) have been developed specifically as one of the IoT’s enabling technologies for long-range applications [10,11,12,13]. LoRa is a PHY-layer LPWAN technology that provides long-range communication at low data rates. Due to its scalability, low power consumption, and ease of deployment, LoRa technology has gained a lot of attention from researchers recently. Its attributes make it suitable for IoT applications, particularly when used as part of LoRaWAN—a protocol used to create a star topology network using LoRa technology. However, when it comes to MANETs and FANETs, LoRaWAN presents some limitations regarding its star topology, its medium access control (MAC) layer and its lack of routing procedures [14].

Some work has been done to assess the performance of LoRa without the constraints of LoRaWAN, for static and ground-mobile ad hoc mesh networks in typical IoT scenarios [15,16,17,18,19,20,21,22,23]. However, there is little research activity on FANETs using LoRa technology. Therefore, a systematic review is needed to determine the technological maturity of FANETs using LoRa and to understand the requirements for the MAC protocols and routing mechanisms associated with such implementation. Moreover, FANETs have particular mobility characteristics when compared to a usual MANET; thus, a review of mobility models and optimal placement algorithms is also required for this kind of network.

### 1.2. Methodology

A systematic literature review was conducted aiming at finding the most common or, if possible, more appropriate MAC protocols and routing mechanisms used for the deployment of LoRa-based FANETs. Furthermore, the review also aims at identifying the mobility or optimal positioning models that have been used, and the role LoRa plays in these works.

#### 1.2.1. Search Strategy

The search was performed using the Google Scholar search engine and the following digital libraries: IEEE Xplore, Multidisciplinary Digital Publishing Institute (MDPI), ScienceDirect, and the Association for Computing Machinery (ACM). The keywords and search expressions used are shown in Table 1.

The search was restricted to the English language, and filters for publication year were applied to limit the search results to the last five years (2017–2022), for two reasons: First, LoRaWAN v1.0 was released in 2015, bringing attention from various researchers and industry; therefore, a multitude of research and publication of articles on related topics has occurred from 2017 onwards. Second, the goal was to review the most recent literature in this field.

#### 1.2.2. Inclusion and Exclusion Criteria

In order to limit the included articles to a more manageable number, they were arranged by relevance, and only the most relevant per search expression were selected from each source library after screening titles and abstracts, with a limit of three when available. After a more thorough review of the retrieved articles, additional publications were included from their references when identified as relevant.

### 1.3. Scope

Numerous studies have been carried out using LoRa or LoRaWAN with UAVs, but not many have covered the complete spectrum of topics involved in the deployment of FANETs. The identified problems involved in a FANET implementation are related to communications, mobility, and energy constraints, and are listed in Table 2.

The specified problems are inherent to FANETs, regardless of the purpose that LoRa serves in the network, whether it is as a backhaul network, access network, or both. It is important to identify how these topics are addressed when LoRa is used in the context of FANETs.

The contributions of this paper are listed as follows:

- Providing a breakdown and discussion of the research challenges involved in the implementation of FANETs.
- Exploring the state of the art of using LoRa technology in FANETs.
- Identifying the mobility models, MAC protocols, and routing techniques that are commonly used for the implementation of FANETs using LoRa technology.

The remainder of the paper is organized as follows: Section 2 presents an overview of LoRa and FANETs. Section 3 covers the critical review of the literature in the context of communications, mobility, and energy constraints. A discussion of the findings and open issues is contained in Section 4. Finally, the concluding remarks are stated in Section 5.

## 2. Overview of LoRa and FANETs

The radio access network for IoT devices has particular requirements: scalability, low cost, long-range coverage, and low power consumption. Many competing technologies intend to provide radio network access for the IoT. There are also several LPWAN technologies, such as Ingenu, Weightless (W, N and P), Sigfox, and LoRaWAN [10,12]. Out of all of these, LoRaWAN is one of the most adopted, because of its relative simplicity and low cost [10,24].

Among cellular solutions for IoT access networks, we can count the Third Generation Partnership Project (3GPP) standards as EC-GSM-IoT, Narrow Band IoT (NB-IoT), enhanced Machine-Type Communications (eMTC) [25], and Massive Machine-Type Communications (mMTC), which is the current IoT specification in 5G (Releases 16 and 17) [26]. These specifications have managed to reduce costs and energy consumption but have not been able to reach the adoption levels of other LPWAN technologies [10,13].

The same features that make LoRa suitable for IoT access networks also make it appealing for its use in FANET communications, which are of mesh topology in nature. Battery constraints limit the flight time of UAVs; thus, a communication mechanism that requires less energy is convenient. Also, one of FANETs potential applications derives from situations where conventional communications infrastructure might be unavailable, in which case long-range capabilities are desirable. In this scenario, exploiting LoRa, in combination with medium access control and routing mechanisms, is more adequate than using LoRaWAN, which has a star topology. A technical background on LoRa and LoRaWAN is presented in Section 2.1, while a description of FANET characteristics is presented in Section 2.2.

### 2.1. LoRa and LoRaWAN

Often, LoRa and LoRaWAN are mentioned interchangeably; however, though complementary, they are two different things. LoRa is a PHY layer proprietary technology owned by Semtech [27], while LoRaWAN is an open network protocol specification—promoted by the LoRa Alliance—that uses LoRa as its physical layer but includes MAC and application layers [27,28].

#### 2.1.1. LoRa

LoRa uses a form of spread spectrum modulation called Chirp Spread Spectrum (CSS) to achieve low-power communications in the range of kilometers [13,29,30] at the expense of data rate. In this modulation technique, symbols are made of chirps. Chirps are sinusoidal signals whose frequency increases or decreases continuously within a certain range and at a certain rate [31]. The modulation parameters are described next and summarized in Table 3.

A. Frequency

LoRa was conceived to transmit over unlicensed spectrum in industrial, scientific, and medical (ISM) bands. It currently operates in the 169 MHz, 433 MHz, 470 MHz, 490 MHz, 780 MHz, 868 MHz, 915 MHz, and 2.4 GHz bands [32], subject to national and regional regulations.

B. Bandwidth (BW)

Bandwidth is the frequency range over which the chirps vary. It can take any of ten values ranging from 7.8 kHz to 1625 kHz, depending on the chipset and frequency band [33,34,35,36,37].

C. Spreading Factor (SF)

The spreading factor represents the rate at which the frequency varies over the bandwidth. In other words, it defines the chirp (symbol) duration. The SF currently ranges from 5 to 12 [35,36,37], and the relationship between the SF value and the symbol duration is defined as follows:

(1) $$T_{s}=\frac{2^{SF}}{BW\left[Hz\right]}\left[s\right],$$

where $T_{S}$ is the symbol duration. Reciprocally, the symbol rate can be defined as:

(2) $$R_{s}=\frac{BW\left[Hz\right]}{2^{SF}}\left[symbols/s\right].$$

According to the LoRa design, the SF also represents the number of modulated bits per symbol, through which we can obtain the modulated bit rate:

(3) $$R_{m}=SF\times R_{S}=SF\times \frac{BW\left[Hz\right]}{2^{SF}}\left[bits/s\right].$$

Considering that each symbol has the same duration, this tells us that the symbols are defined by the starting frequency of the chirp.

D. Coding Rate (CR)

LoRa implements forward error correction (FEC) by adding redundancy bits to every four bits of data. The number of redundancy bits is given by CR and can go from one to four. Thus, ref. [31] defines the *rate code* as:

(4) $$Rate\;Code=\frac{4}{4+CR}.$$

The data bit rate is the product of the modulated bit rate and the *rate code*, as follows:

(5) $$R_{b}=R_{m}\times RateCode=SF\times \frac{BW\left[Hz\right]}{2^{SF}}\times \frac{4}{4+CR}\left[bits/s\right].$$

E. Transmission Power

The transmission power can reach up to 22 dBm, depending on the chipset selection and power amplifier configuration [33,34,35,36,37].

Two of the key parameters behind LoRa modulation are SF and BW. The relationship between these two factors defines the signal’s data rate, range, and time on air. The higher the SF, the lower the transmission rate and the longer the range. Conversely, the lower the SF, the higher the transmission rate and the shorter the range.

**Table 3:** Summary of LoRa modulation parameters.

Parameter	Magnitude/Range	Chip	Reference
Frequency	137–175 MHz	SX1276/77/78/79	[33]
	410–525 MHz	SX1276/77/78/79	[33]
	862–1020 MHz	SX1276/77/79	[33]
	860–1020 MHz	SX1272/73	[34]
	410–810 MHz	SX1268	[35]
	150–960 MHz	SX1261/2	[36]
	2.4 GHz	SX1280/SX1281	[37]
Bandwidth (BW)	7.8 kHz	SX1276/77/78/79, SX1268, SX1261/2	[33,35,36]
	10.4 kHz	SX1276/77/78/79, SX1268, SX1261/2	[33,35,36]
	15.6 kHz	SX1276/77/78/79, SX1268, SX1261/2	[33,35,36]
	20.8 kHz	SX1276/77/78/79, SX1268, SX1261/2	[33,35,36]
	31.2 kHz	SX1276/77/78/79, SX1268, SX1261/2	[33,35,36]
	41.7 kHz	SX1276/77/78/79, SX1268, SX1261/2	[33,35,36]
	62.5 kHz	SX1276/77/78/79, SX1268, SX1261/2	[33,35,36]
	125 kHz	SX1276/77/78/79, SX1272/73, SX1268, SX1261/2	[33,34,35,36]
	250 kHz	SX1276/77/78/79, SX1272/73, SX1268, SX1261/2	[33,34,35,36]
	500 kHz	SX1276/77/78/79, SX1272/73, SX1268, SX1261/2	[33,34,35,36]
	203 kHz	SX1280/SX1281	[37]
	406 kHz	SX1280/SX1281	[37]
	812 kHz	SX1280/SX1281	[37]
	1625 kHz	SX1280/SX1281	[37]
Spreading Factor (SF)	5	SX1268, SX1261/2, SX1280/SX1281	[35,36,37]
	6–9	SX1276/77/78/79, SX1272/73, SX1268, SX1261/2, SX1280/SX1281	[33,34,35,36,37]
	10–12	SX1276/78/79, SX1272, SX1268, SX1261/2, SX1280/SX1281	[33,34,35,36,37]
Coding Rate (CR)	1 (4/5)	SX1276/77/78/79, SX1272/73, SX1268, SX1261/2, SX1280/SX1281	[33,34,35,36,37]
	2 (4/6)	SX1276/77/78/79, SX1272/73, SX1268, SX1261/2, SX1280/SX1281	[33,34,35,36,37]
	3 (4/7)	SX1276/77/78/79, SX1272/73, SX1268, SX1261/2, SX1280/SX1281	[33,34,35,36,37]
	4 (4/8)	SX1276/77/78/79, SX1272/73, SX1268, SX1261/2, SX1280/SX1281	[33,34,35,36,37]
Transmission Power	−4 to 20 dBm	SX1276/77/78/79	[33]
	−1 to 20 dBm	SX1272/73	[34]
	−17 to 22 dBm	SX1268	[35]
	−17 to 22 dBm	SX1261/2	[36]
	−18 to 12.5 dBm	SX1280/SX1281	[37]

#### 2.1.2. LoRaWAN

LoRaWAN is an open network specification built on top of LoRa and developed to be used by battery-powered sensors that send data over long distances at low data rates [27]. It defines the system architecture of the network and its communication protocol.

A. Architecture

LoRaWAN defines a hierarchical network architecture with the following elements:

- End devices: Also called nodes; they are usually sensors, actuators, or both, equipped with LoRa transceivers that connect to one or more gateways in a single hop.
- Gateways: They connect the LoRa access network to any standard IP backhaul network to relay data between the end nodes and the network server.
- Network server: It is in charge of routing the data between the end device and the appropriate application server. It also handles network layer security by using AES-128 encryption to authenticate end devices.
- Application server: It manages the application to which the end device data is aimed. It processes the data, presents it to the user, and replies to the end device, if necessary. It also handles application layer security by using AES-128 encryption to keep the end user’s application data confidential to the network operator.

**Figure 1:**
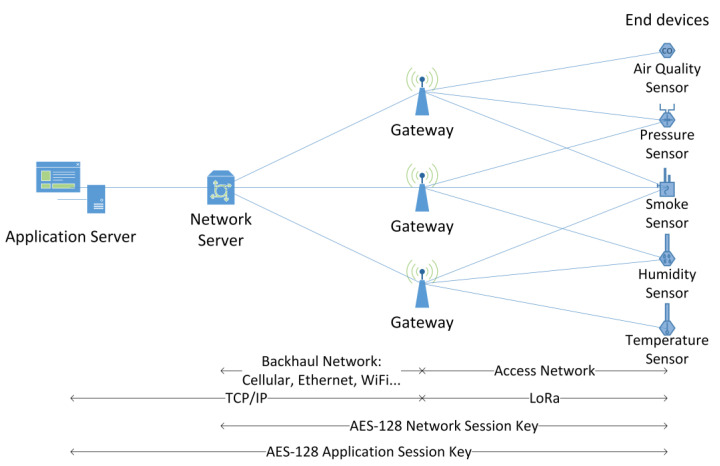
LoRaWAN architecture.

These network elements typically connect in a star-of-stars topology as shown in Figure 1.

B. Communications

As mentioned before, the LoRaWAN specification establishes the use of LoRa at its physical layer [27]. Additionally, LoRaWAN describes MAC and application layers whose implementation depends to some extent on the end device’s class, which can be one of three classes as explained below:

- Class A: The end devices of this class are half-duplex transceivers that implement pure ALOHA for their uplink transmissions, meaning that they transmit when they need to do it, but only after a small random time has elapsed. The receiver remains off, except for two receive windows that open after an uplink transmission. This is the class with the lowest energy consumption, and all LoRaWAN end devices must implement it.
- Class B: This class is meant for applications in which the end device needs to download more traffic than Class A devices. End devices of this class employ all functionalities of Class A, but open additional reception windows (also called ping slots) in a scheduled manner. For these reception windows to work, synchronization is required, which is achieved by the gateway sending periodic beacons to all end nodes.
- Class C: End devices of this class listen continuously except when they are transmitting. As this is the class with the most energy consumption, it is meant for applications that are less power-constrained. End devices of this class also implement all functionalities of Class A but must not enable Class B concurrently.

**Figure 2:**
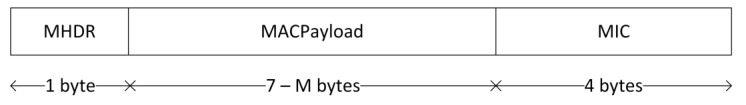
LoRaWAN frame.

The LoRaWAN specification also defines MAC frame formats. As with any protocol data unit, frame formats are intended to allow communication between peer elements in a layered network, for which they separate the protocol control information (overhead) from the payload, in a standardized way. The LoRaWAN frame, which is the payload for the LoRa physical layer, has three fields as shown in Figure 2. The first field is the MAC header (MHDR), which is 1 byte long and specifies the type of frame. The second field is the MAC Payload, whose length can range from 7 to *M* bytes, where the maximum size of *M* can reach up to 250 bytes and is calculated based on the maximum allowed LoRa transmission time [38]. Finally, the frame closes with a 4-byte message integrity code (MIC), which is calculated over all the fields in the frame.

Regarding control information, a series of MAC commands are used to exchange control information exclusively between the network server and the end devices. As all end devices join the network in Class A mode, Class B and Class C devices must implement all Class A MAC commands.

To be part of a LoRaWAN network, every end device must first be activated following one of the next two methods:

- Activation by Personalization: The information required by the end device to join a network is statically stored in it. This method is technically simpler, requires access to the end device, and is intended to be used mostly in private networks.
- Over-the-Air Activation: The end device initiates a join procedure by sending an unencrypted Join Request frame to the network server. If the Join Request is accepted, the server responds with an encrypted Join Accept frame. This method is dynamic and can be used in public or private networks.

### 2.2. Flying Ad Hoc Networks (FANETs)

**Figure 3:**
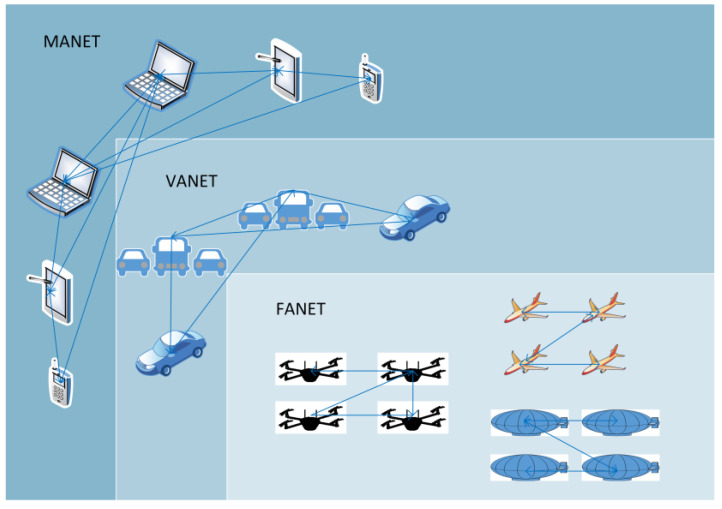
Relationship between MANET, VANET and FANET.

Although UAVs have been used in the military since World War I [39,40], it is not until recently that they have become widely available for civilian applications, such as 3D mapping, construction inspection, land surveying, oil exploration, agricultural monitoring, emergency response operations, surveillance, and asset management, to name a few. Many of these applications could benefit from using a swarm of drones instead of a single UAV, for which an ad hoc network could be established for communication and coordination. The authors of [41] consider FANETs as a subset of MANETs but not as a subset of Vehicular Ad Hoc Networks (VANETs), while others [42,43,44,45] also consider FANETs to be a subset of VANETs. Furthermore, Bekmezci et al. [44] define a FANET as a form of MANET made of multiple UAV nodes, where the communication between UAVs cannot rely on infrastructure-based links. The relationship between MANETs, VANETs, and FANETs is illustrated in Figure 3.

#### 2.2.1. UAV Taxonomy

Regarding UAV taxonomy, multiple categorizations are proposed in [41,42,43]. A summary of this topic is presented in Table 4.

#### 2.2.2. Differences between FANETs, VANETs, and MANETs

The differences between FANETs, VANETs, and MANETs are analyzed from different perspectives in [41,42,43,44,45] and presented in Table 5. The aforementioned works highlight the fact that FANETs have specific characteristics. Those specificities are summarized in the following fields:

- Node mobility: Contrary to the elements of MANETs and ground VANETs, UAVs experience relatively fewer obstacles, which allows them to move in and around three axes with a certain amount of freedom at somewhat constant speeds. However, holding a fixed position can be more challenging, or even impossible, depending on weather conditions and the type of UAV. These circumstances influence the mobility model to be applied but also impact other characteristics, such as node density, topology change rate, localization alternatives, and applicable propagation models.
- Radio propagation: The presence of fewer obstacles allows for the consideration of line-of-sight (LoS) propagation while taking into account weather conditions and the Doppler effect caused by the speed of UAVs relative to the ground and to one another. Air-to-air and air-to-ground are the two main types of links that can be identified, although air-to-satellite links might also be considered for some applications.
- Energy constraints: They depend on the type of UAV. Battery-powered UAVs are more energy-constrained, making it useful to have communication hardware that consumes less power, allowing for increased flight time, although most of the energy is dedicated to keeping the UAV and its payload in the air. Large fixed-wing UAVs are most likely powered by combustion engines that can carry and charge larger batteries, making them less energy-constrained.

**Table 5:** Differences between FANET, VANET and MANET.

Characteristic	MANET	VANET (Ground)	FANET
Elements [45]	Mobile phones [45]	Vehicles [45]	UAVs, airplanes [45], balloons, HAPs
Node speed [41,42,45]	6 km/h [41,42] 0–1.5 m/s [45]	20–130 km/h [41] 20–100 km/h [42] 4–36 m/s [45]	6–460 km/h [41] 50–100 km/h [42] 8–257 m/s [45]
Node mobility [41,42,43,44]	Consistent, 2D, Random trajectories, Low [41] Lower (2D) [42] Relatively slow compared to VANET and FANET [44]	Consistent, 2D, Random trajectories, High [41] Low (2D) [42]	Free, 3D, Either random or predefined trajectories, very high [41] Medium to high (3D) [42] Faster than MANET and VANET [43] Much higher than MANET and VANET [44]
Node density [43,44,45]	Dense [45]	Dense in cities and sparse in rural areas [45]	Much lower than MANET and VANET [43,44] Mission-dependent [45]
Mobility model [41,42,43,44]	Random [42] Random Way Point (RWP) [44]	Manhattan Models [42] High predictability [44]	RWP, Paparazzi (PPRZM) [41,42] Predetermined, random [43] Predetermined, random, Semi-Random Circular Movement (SRCM), Pheromone map [44]
Topology change [41,42,43,44]	Dynamic, unpredictable [41] Low [42]	Linear movement but more progressive than VANET [41] Medium [42]	Stationary, Slow and Fast [41] High [42] More frequent than MANET or VANET and related to node mobility/availability [43]
Propagation model [41,42,43,44,45]	Non-Line-of-Sight (NLoS) [41,44] Rayleigh [45]	Non-Line-of-Sight (NLoS) [41,44] Rayleigh/Rician [45]	Line-of-Sight (LoS) [41,43,44] Friis, Rice, Log-Normal [42] Rayleigh/Rician [45]
Energy constraints [41,42,43,44,45]	Medium [41,42] Constraint [45]	Low [41,42] Non-constraint [45]	Medium to High [41,42] Low to High depending on the type of UAV [43] High for mini-UAVs [44] Constraint/ Non-constraint [45]
Computational power [41,44]	Limited [41,44]	High [41,44]	High [41,44]
Localization [41,43,44]	Global Positioning System (GPS) [41,44]	GPS/Assisted GPS (AGPS) [41,44] Differential GPS (DGPS) [44]	GPS/AGPS (Assisted Global Positioning System) [41,44] DGPS, Inertial Measurement Unit (IMU) [44]

## 3. Critical Review

The critical review will be covered according to the identified challenges involved in a FANET implementation as stated in Section 1.3.

### 3.1. Communications

The communication challenges can be generally described under the conceptual framework of the Open Systems Interconnection (OSI) or TCP/IP layer models, while taking into consideration the mobility and energy constraints of FANETs. The PHY layer will be limited to LoRa and LoRaWAN, which are within the scope of this work.

#### 3.1.1. Architecture

The communication architecture is highly dependent on the network application. First, a common ground for FANETs must be determined, and then, some architectures are exemplified to establish the types of applications on which they are employed. Single UAV architectures are not considered as FANETs because FANETs are composed of more than one UAV, and communication between UAVs cannot rely on infrastructure networks [44]. Moreover, this means that topologies can be mesh, star-of-meshes, or mesh-of-meshes.

**Figure 4:**
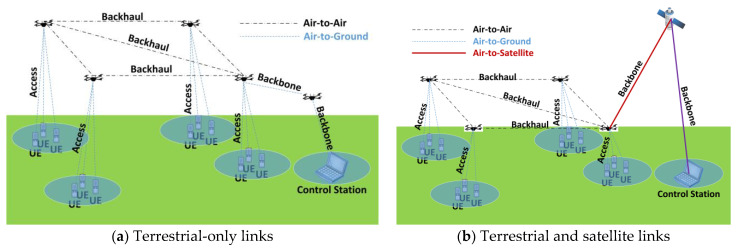
FANET types of links.

As mentioned in Section 2.2.2, three different types of links can be identified according to the locations of the elements they connect, namely air-to-air (UAV-to-UAV), air-to-ground (UAV-to-ground) and air-to-satellite (UAV-to-satellite). Links can also be classified according to the role they play from a communications network perspective: access links, backhaul links, and backbone links. The two mentioned classifications are illustrated in Figure 4.

Four UAV communication architectures are mentioned in [43], based on the type of infrastructure utilized: UAV direct communication, UAV communication via satellite networks, UAV communication via cellular networks, and UAV communication via ad hoc networks. However, these could be further summarized into communication through infrastructure and infrastructure-less communication; hence, only UAV communication via ad hoc networks corresponds to FANETs.

Three hierarchical architectures are described in [46,47], based on how the UAVs connect to each other and to a ground base station. The first architecture relies on a single UAV acting as a hub to connect a single group of UAVs to the base station. The second architecture involves clustering UAVs into groups, each one of them having one hub to connect to the base station. Finally, in the third architecture, multiple layers of UAV clusters connect to each other through one root UAV, and only one of the groups has a hub that connects all others to the base station. According to the description, UAV-to-UAV communication relies on low-power, short-range links, while UAV-to-ground communication does it on high-power, long-range links. These architectures could be employed in applications wherein compact swarms with longer UAV-to-ground ranges—when compared to UAV-to-UAV ranges—are needed. Moreover, having centralized links to the base station implies one or more of the following situations:

- Most communications take place inside the swarms.
- The communication with the base station is less frequent or takes place at low data rates.
- The UAV that handles the link to the base station is a single point of failure and may become a bottleneck.

**Figure 5:**
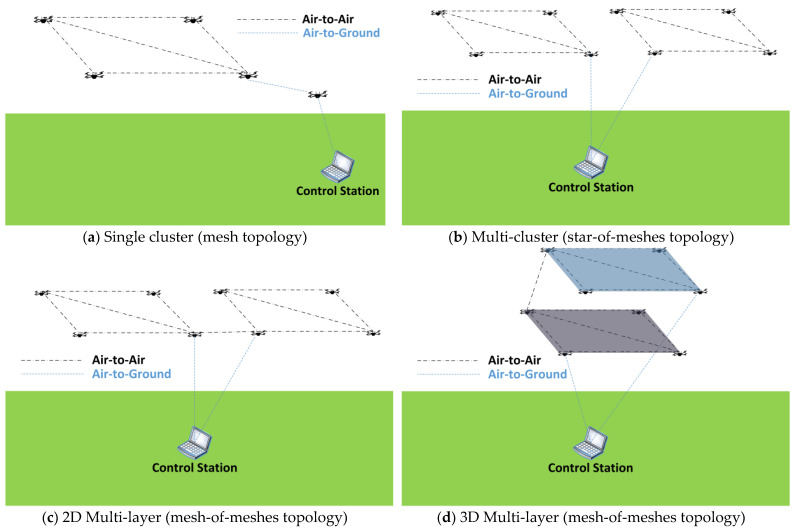
FANET topologies.

To overcome the last issue, the authors of [48] propose a multi-layer architecture where clusters are grouped in layers and each cluster selects a UAV that acts as a hub to connect to the base station or to another layer, and a second UAV as a backup hub. The UAV clusters can be grouped two-dimensionally or in multiple layers in the three-dimensional space. A similar multi-layer approach is also presented in [49]. The architectures described in [46,47,48,49] can be synthesized by topology into the categories shown in Figure 5.

**Figure 6:**
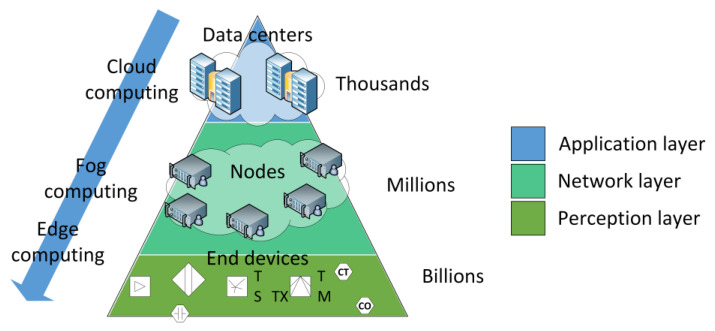
Superposition of IoT and cloud architectures.

In the case of FANETs being a part of an IoT implementation, architectures can be local or cloud-based. When cloud infrastructure is involved, a superposition of the IoT three-layered architectural model [50] and the IoT-oriented cloud computing architecture can be useful to understand the FANET role. The IoT may present the need to have computing resources closer to the end devices, which has led to the coining of the terms *edge computing* and *fog computing*. Cloud, fog, and edge computing refer to the segment where the computations are performed with respect to the distance from the end node, as shown in Figure 6. Furthermore, the end device could be mounted on the ground or on the UAV, depending on the application. In both cases, the UAV becomes an edge computing enabler.

Regarding the use of LoRa or LoRaWAN, most works refer to UAV-aided wireless sensor networks (WSNs) with single or independent UAVs [51,52,53,54,55,56,57,58]. WSNs are sets of sensors that communicate with each other by forming an ad hoc network. By this definition, WSNs can be considered a subset of the IoT. However, even though WSNs and FANETs have a mesh nature in common, a WSN aided by a single UAV is not considered a FANET by the definition presented in Section 2.2.

A system called LoRaUAV is proposed in [59,60] aimed at providing coverage to mobile sensors during emergencies by deploying a mesh of LoRaWAN gateways mounted on UAVs. In this work, LoRaWAN is used as an access network for a fixed number of ground-mobile sensors, while UAVs relay information through a Wi-Fi mesh network to a base station. In this architecture, the LoRaWAN access network range is longer than that of the Wi-Fi mesh backhaul.

The feasibility of a hybrid architecture made of LoRa and IEEE 802.11 s overlaid mesh networks is analyzed in [61], where LoRa and LoRaWAN are proposed for long-range-low-data-rate communications and IEEE 802.11 s for short-to-mid-range-high-data-rate communications. A hierarchical tree of multi-layer meshes is suggested, where a smart selection mechanism switches between IEEE 802.11 s and pure LoRa for UAV-to-UAV communications, and between IEEE 802.11 s and LoRaWAN for UAV-to-ground communications. The proposed architecture is aimed at being a general approach for a wide range of UAV-based scenarios, where, given its mobility and radio propagation conditions, a combination of long-range-low-data-rate and short-range-high-data-rate protocols might be convenient.

A single-cluster mesh is the system topology presented in [62]. The network has sensor nodes, relay nodes (mounted on UAVs), and a gateway on the ground, where the gateway operates as the root node of the mesh network, providing the air-to-ground backbone link.

A summary of the literature findings regarding proposed architectures and their applications is shown in Table 6.

#### 3.1.2. Medium Access Control

**Figure 7:**
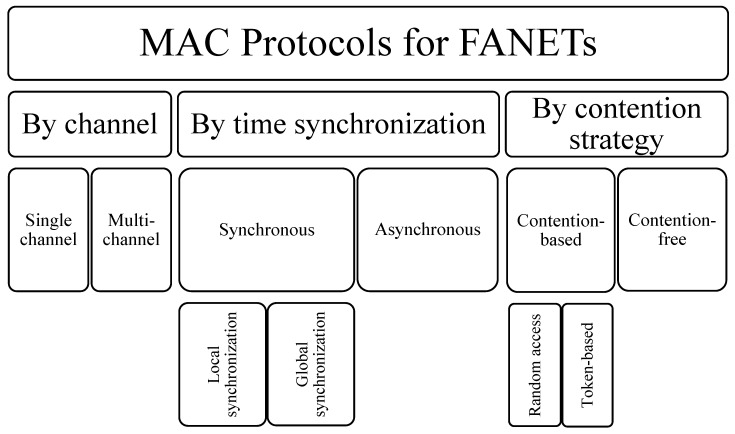
Taxonomy of MAC protocols for FANETs.

MAC protocols coordinate access to a shared medium with the purpose of avoiding collisions in an efficient, fair, and timely manner. As with all other topics pertaining to FANET communication, MAC protocols must be chosen or designed to fulfill their goal while considering the corresponding mobility characteristics and energy restrictions of FANETs. A classification of MAC protocols can be helpful in deciding which one to use for a particular application; however, this can be a demanding task because of the many parameters that can be used to characterize them [64]. A taxonomy of MAC protocols for WSNs is presented in [65], where four condensed categories are discussed: asynchronous, synchronous, frame-slotted, and multi-channel. A classification of multi-channel MAC protocols for low-power and lossy networks is proposed in [64], with five main categories defined by channel assignment scheme, medium access strategy, way of interaction with upper layers, MAC layer coordination, and physical layer management. The authors of [66] present a taxonomy of MAC protocols for FANETs with three main categories based on their channel access strategy: contention-based, contention-free, and hybrid. An analysis of MAC protocols for the IoT along with MAC protocols for UAV networks is performed in [67], where the authors also present a classification of MAC protocols for a UAV-based IoT (UIoT) with three categories: contention-based, contention-free, and based on artificial intelligence (AI-based). Starting from these classifications and expanding the summarized categories, a taxonomy of MAC protocols for FANETs is presented in Figure 7.

As seen in Table 6, the works presented in [59,60,61,62,63] consider the use of LoRa or LoRaWAN as part of a multi-UAV solution that involves FANET communication. Moreover, only [61,62,63] consider the use of LoRa at the heart of the FANET, i.e., on the air-to-air links. Therefore, only the last three works propose the use of MAC protocols different than that of LoRaWAN. A summary of the MAC protocols used in FANETs that involve LoRa or LoRaWAN is shown in Table 7.

In order to achieve mesh networking using LoRa, a packet structure and a Time Division Multiple Access (TDMA) scheduling are proposed in [61]. The proposed packet structure includes fields for node identification, node positioning, and mesh configuration data, intended for mesh setup and update. Regarding performance evaluation, the authors present a theoretical analysis of the operating range of both LoRa and IEEE 802.11 s. Also, a simple experimental analysis of the LoRaWAN operating range is performed for UAV-to-ground communications.

Carrier Sense Multiple Access with Collision Avoidance (CSMA/CA) and a customized form of Destination Sequenced Distance Vector (DSDV) routing protocol are assessed in [63] to achieve a decentralized mesh network of flying drones. Two-hop performance is assessed experimentally in terms of channel usage, packet delivery rate, route construction time, and delay.

#### 3.1.3. Routing

There are countless routing protocols and different ways to classify them depending on the context in which they are applied. Various routing schemes for FANETs can be found in [41,43,46,48,68,69,70,71,72,73,74]. A taxonomy of FANET routing protocols is presented in [46], where they are categorized into static, proactive, reactive, hybrid (proactive and reactive), geographic (position-based), and hierarchical. A similar classification is performed in [43], with the difference that it does not include hierarchical routing protocols as a category. Additionally, thorough taxonomies—which reach up to four levels in depth—are provided in [41,70,71,72], where additional categories such as bio-inspired routing, swarm-based routing, and machine-learning-based routing are worth mentioning.

Regarding the use of LoRa or LoRaWAN in FANETs, the same observation made in Section 3.1.2 applies to the present section, with the additional consideration that LoRaWAN does not incorporate routing within its network architecture. The routing approaches presented in [59,60,61,62,63] are discussed next and summarized in Table 8.

Although LoRa is not part of the air-to-air links in [59,60], Optimized Link State Routing (OLSR) is the protocol used within the proposed Wi-Fi mesh network. According to the authors, the mobility mechanism requires knowledge of the full network topology, which is the main consideration behind this decision.

The Hybrid Wireless Mesh Protocol (HWMP)—which is the default routing protocol for 802.11 s—is mentioned as the routing protocol for the Wi-Fi mesh network in [61], while no routing protocols are mentioned for its use with LoRa. A GPS-based directed flooding approach is proposed in [62] as the routing protocol, where an experimental test with one relay node and two sensor nodes (2 hops) is performed.

The authors of [75] propose an improvement to 802.11 s routing metrics for its use in the deployment of FANETs. Claiming that HWMP was not conceived for FANET mobility, this work presents two new routing metrics aimed at improving network performance in terms of throughput and end-to-end delay. More specifically, the first metric is based on modifications to the default frame error rate and link time usage calculations to improve performance for stationary nodes. The second metric adds a factor based on the Received Signal Strength Indicator (RSSI) of a wireless link, increasing its cost when the fade margin falls below a certain threshold.

### 3.2. Mobility

Transportation and communication problems are analogous in many ways. In fact, many terms used in communications—such as packet or collision—come from the postal or transportation worlds. This analogy gives us a sense of the importance of addressing the mobility problem in FANETs. Moreover, mobility can be seen as an opportunity instead of a problem, where nodes can move and reposition themselves to reconfigure a network or to recover connectivity. In this section, the FANET mobility issue is broken down into mobility objectives and mobility models.

#### 3.2.1. Mobility Objectives

The mobility objectives can be described as optimization problems that are dependent on various factors such as application, environmental conditions, and available resources. Optimization can be performed in the following ways:

- Optimal positioning: Where to go and why.
- Optimal trajectory determination: How to get there and why.
- Optimal agent selection: Which UAVs should get there and why.

Regardless of the specific application, a common requirement for FANETs is to maximize network uptime, in the literal and figurative senses of the expression.

#### 3.2.2. Mobility Models

Mobility models can be used to model movement for FANET simulation and to achieve mobility optimization goals in practical implementations. Four mobility models for FANETs are surveyed in [76]: Random Way Point, Gauss-Markov, Semi-random Circular Movement, and Mission Plan Based. The same models are reviewed in [43,68], with the addition of the Pheromone-Based, Paparazzi, and Particle Swarm Mobility Models. Furthermore, a taxonomy of mobility models with five categories is provided in [41], namely, random-based, time-based, path-based, group-based, and topology-based.

Reference Point Group Mobility (RPGM) is used as the mobility model for performance evaluations in [75], which are carried out through simulations in Network Simulator (NS)-3. These simulations show that the inclusion of an RSSI-based factor results in an improvement in network throughput and a decrease in the number of route changes, with little to no impact on end-to-end delay, especially for a mobile scenario.

An algorithm to find the optimal position of a UAV-mounted relay node is presented in [77] to maximize the throughput between any pair of fixed ground nodes within a wireless mesh network. To achieve this, nodes—whose positions are known—are first clustered in a way such that nodes that connect within a certain RSSI margin are part of the same cluster. Then, the placement optimization is performed by separating the positioning problem into horizontal and vertical placement and iterating between the two. For the horizontal placement, clusters are considered single entities around their center of gravity, and the UAV is positioned to maximize throughput—as a function of signal-to-noise ratio—for the worst wireless link. Subsequently, vertical positioning is determined to maximize throughput in the same manner. Because this work focuses on static mesh networks, routing is done by means of an optimized link state routing protocol (OLSRD). Simulations for up to 20 ground nodes are implemented using a 3D map. Also, an experimental performance assessment is presented with four ground nodes separated into two clusters of two nodes each.

Concerning mobility models in FANETs that use LoRa, a Connection Recovery and Maintenance (CRM) algorithm is proposed in [59,60], where a two-dimensional mobility problem is divided into four mobility modes. A Virtual Spring Force (VSF) mobility algorithm is used to handle proximity between nodes to avoid collisions and to maintain distance within communications range. Correspondingly, if the forces are in balance, the UAV goes into stationary mode. Then, if a path to the base station is lost, the UAV goes into network recovery mode, where it moves in the direction of the base station; and, if the connection is lost with a ground node, movement prediction is used alongside the spring force mobility to try to reestablish it, based on the ground node’s last known position, direction, and speed.

### 3.3. Energy

Considering that large fixed-wing UAVs are less energy-constrained, this section focuses on FANETs comprised of battery-powered UAVs. In energy-constrained FANETs, energy consumption efficiency may be achieved by taking one or more of the following measures [78]:

- Reducing UAV and payload weight.
- Increasing aerodynamic efficiency.
- Improving communication protocols, or selecting them, for reduced energy consumption.
- Optimizing motion to reduce energy consumption.

Even though they do not necessarily increase energy consumption efficiency, in-air energy replenishment procedures might be considered to maximize flight time, such as the use of solar panels or the implementation of radio energy harvesting.

Battery-powered UAVs are constituted by many active communication and mobility elements, e.g., radio transceivers, Global Navigation Satellite System (GNSS) units, flight controllers, motors, cameras, and other sensors. Although a lot of work focuses on developing energy-efficient MAC and routing protocols [79,80,81,82,83,84,85,86,87], the energy required for mobility is much higher than the energy required for communications [88]. To exemplify the last statement, a conservative energy consumption calculation is shown in Table 9 for a hybrid LoRa/Wi-Fi UAV network node, assuming that the transmitters work continuously at maximum power during a 30-min flight.

The payload weight and the percentage of energy dedicated to communications can be reduced if the control hardware includes a Wi-Fi transceiver, which is the case with the Raspberry Pi, as well as with many other single board computers (SBC) and system-on-a-chip microcontrollers. This information suggests that it might be more significant to focus on taking measures to optimize energy consumption from the mobility perspective. From the communications standpoint, this means that efforts should be focused on the reduction of the weight and size of its components, something that is already being done.

Regarding the use of mechanisms to collect energy during flight, the performance of radio frequency (RF) energy harvesting in a UAV Relay Assisted IoT Communication Network is assessed in [93]. In this study, time division and power splitting schemes are implemented to perform communications reception, transmission, and energy harvesting. The system assessment is done through simulation, using communications metrics like system throughput, outage probability (availability), and bit error rate (BER). Although no information is provided regarding the amount of energy collected or saved, it can be inferred that the system aims at reducing the transmission power of IoT sensors, which, as mentioned earlier, might not have a significant impact on increasing UAV flight time. A similar conclusion can be drawn from [94]. On the other hand, a solar energy-harvesting model is proposed in [95] to power small UAVs, where analytical results show possible flight time improvements in the range of 3 to 50 min, depending on the flight conditions.

## 4. Discussion on Findings and Open Issues

The systematic literature review shows that LoRa is mostly used in single UAV architectures for WSNs [51,52,53,54,55,56,57,58]. A few works have considered LoRa or LoRaWAN as part of a FANET implementation using hybrid architectures combined with Wi-Fi [59,60,61], whereas some other works have explored the application of MAC and routing protocols to extend the use of LoRa to a UAV mesh network [62,63]. This might be attributed in part to the fact that an accurate assessment of network performance requires a multidisciplinary implementation of mobility models and complete network stack implementation, even if simplified or abstracted. The rest of the findings and open issues are addressed in accordance with communication, mobility, and energy aspects. Additionally, Table 10 presents a comparison of FANET-related characteristics between LoRa/LoRaWAN, Wi-Fi, and cellular LPWANs.

### 4.1. Communications

Regardless of LoRa data rate limitations, its communication range can be exploited for applications that require maximum distance between UAVs. Using LoRa as a FANET access network might limit applications to WSN or flight control, considering that LoRa devices are not as extended among end users as those of other types of communication. Therefore, a hybrid network could be more convenient for human-oriented communications, in applications such as localization, short messaging, or search and rescue operations, where the access network should be of widespread use, such as Wi-Fi or cellular. For future research, it will be important to test the maximum communication ranges at the maximum data rate available for the latest LoRa chips in the 2.4 GHz band, which might enable some real-time applications.

As mentioned in Section 3.3, efforts in communications improvement could be aimed at reducing equipment weight and improving air-to-air range, rather than at reducing energy consumption at the expense of network performance. The same consideration applies to network security, where the issues are the same as those of any wireless network, with the exception that the communications equipment could potentially be accessed by anyone that has the UAVs within sight. There is room for developing communication protocols at the physical layer to find a better balance between data rate and communications range, without having to compromise energy consumption excessively.

### 4.2. Mobility

It is essential to handle the mobility of UAVs, as it has a direct impact on FANETs’ communication and energy performance. Mobility models where each UAV moves independently do not seem appropriate for FANETs. On the contrary, group mobility models appear more useful, since network information such as position or received signal power can be shared among multiple nodes. With respect to network stability, mobility plays a crucial role in network reconfiguration, considering that a node can reposition itself to maintain or recover a link. In this case, energy consumption should be considered, but it can be achieved efficiently by keeping in mind that the received power is exponentially and inversely proportional to the distance between nodes, meaning that a small, distance-closing movement can result in the recovery of a link. Hence, mobility models that keep a record of their current, past, or best positions can also be applicable in this context.

The mobility optimization problems must be comprised of more than one objective; namely, the preservation of communications performance indicators such as received signal power or signal-to-noise ratio for air-to-ground and air-to-air links, as well as energy consumption minimization. Additionally, it is necessary to handle network handover between UAVs when they need to be replaced.

Apart from the study in [59,60], related works that involve LoRa do not consider mobility. Again, to assess the impact of mobility models on communications performance, simulations require complete network stack implementation, while real experiments require a swarm of drones with communication equipment.

### 4.3. Energy

Concerning energy consumption, reducing UAV and payload weight seems to be more significant than coming up with communication protocols for improved energy efficiency.

Regarding in-flight energy collection mechanisms, radio energy harvesting may gather enough energy to charge a sensor battery, but it does not generate enough energy to charge a drone considerably. However, solar panels can be an alternative as long as they do not increase UAV weight beyond efficient levels.

Table 11 and Table 12 present a summary of the advantages and disadvantages of using LoRa in FANETs.

## 5. Conclusions

This work has presented a review of LoRa and its usage in the context of Flying Ad Hoc Networks. FANETs have a wide range of potential applications, wherein one of the main design considerations is to find a balance between data rate, communications range, mobility, and energy consumption. Studying this type of network is challenging because the communications, mobility, and energy dimensions must be evaluated concurrently to obtain accurate and significant results. Consequently, end-to-end simulations require full network stack implementation, while practical experiments require a swarm of drones. Though not much research work has been conducted on using LoRa as a mesh backhaul for air-to-air links, this technology can be useful to maximize the communications range between UAVs in low-data-rate applications such as WSN, remote control, flight coordination, or drone identification. Furthermore, the use of hybrid architectures, in which LoRa is combined with more ubiquitous and higher-data-rate technologies, expands its usefulness to applications such as search and rescue as well as short messaging. There is also room for developing communication protocols at the physical layer to achieve increased data rates (when compared to LoRa) while maintaining a long communication range. This means that energy utilization would be increased, but as LoRa is at the lower end of energy consumption, an increase could be possible without affecting UAV flight time considerably. Regarding communication protocols above the physical layer, routing must respond to the topology changes associated with a highly mobile network to avoid unnecessary UAV movement. At the same time, mobility algorithms must optimize positioning, trajectory, and UAV selection, with the purpose of maximizing flight time and network performance while minimizing energy consumption. Finally, the energy harvesting mechanisms must be developed and selected in such a way that they do not affect drone mobility.

## Figures and Tables

**Table 1:** Keywords and search expressions.

No.	Keywords
1.	“LoRa” AND “FANET”
2.	“FANET” AND “Communications”
3.	“FANET” AND “Mobility”
4.	“FANET” AND “Energy”
5.	“LoRa” AND “UAV” AND “Communications”
6.	“LoRa” AND “UAV” AND “Architecture”
7.	“LoRa” AND “UAV” AND (“Medium Access Control” OR “MAC” OR “Mesh”)
8.	“LoRa” AND “UAV” AND “Routing”
9.	“LoRa” AND “UAV” AND “Mobility”
10.	“LoRa” AND “UAV” AND “Energy”

**Table 2:** Challenges involved in a FANET implementation.

Challenge	Topic
Communications	Architecture PHY layer technology Medium access control Routing
Mobility	Mobility objectives Mobility models
Energy	Energy consumption reduction Energy harvesting

**Table 4:** UAV taxonomy.

By Wing Type			By Size		By Type of Flight		By Flight Range				By Energy Autonomy			By Altitude			By Purpose							
Fixed wing [41,42,43]	Rotary wing [41,42,43]	Hybrid [41]	Large [42,43]	Small [42,43]	Autonomous [42]	Remotely controlled [42]	Close-range [43]	Short-range [43]	Mid-range [43]	Long-range	High [42]	Medium [42]	Low [42]	High [42]	Medium [42]	Low [42]	Military [41]	Communications [41,42]	Surveillance [41]	Photography/Mapping [41,43]	Exploration/Surveying [41]	Remote sensing [41]	Delivery [43]	First-Person View (FPV)/Entertainment

**Table 6:** Single UAV and FANET architectures involving LoRa or LoRaWAN.

Reference	Topology	Communication Technology			IoT Architecture	UAV Type	Application	Test
		Air-to-Ground	Air-to-Air	Air-to-Satellite				
[51]	Single UAV	LoRaWAN (access) 4G/Wi-Fi (backbone)	---	---	Edge	Rotary wing	Agricultural monitoring	Single UAV-to-Ground proof of concept
[52]	Single UAV	LoRaWAN/Bluetooth (both as access) Wi-Fi (backbone)	---	---	Edge	Rotary wing	Search and rescue	Single UAV-to-Ground proof of concept
[53]	Single UAV	LoRaWAN (access/backbone)	---	---	Fog	Rotary wing	Disaster monitoring	Single UAV-to-Ground proof of concept
[54]	Single UAV	LoRaWAN (access)	---	Simulation through delay (backbone)	Cloud	Rotary wing	Sensor monitoring	Single UAV-to-Ground proof of concept
[55]	Single UAV	LoRaWAN (access) Wi-Fi (backbone)	---	---	Cloud	Rotary wing	Environmental monitoring	Single UAV-to-Ground proof of concept
[56]	Single UAV	LoRa (access)	---	---	Cloud	---	UAV remote identification	Single UAV-to-Ground simulation
[57]	Multiple independent UAVs	LoRaWAN (access)	---	---	Cloud	Rotary wing	Search and rescue, asset localization	Single UAV-to-Ground experiments
[58]	Single UAV	LoRa (access)	---	---	Cloud	Rotary wing	Sensor monitoring	Single UAV-to-Ground experiments
[59,60]	Single cluster	LoRaWAN (access) 802.11 g (backbone)	802.11 g (backhaul)	---	Cloud	Rotary wing	Emergency response	Simulation
[61]	3D multi-layer	802.11 s/LoRaWAN (both as access/backbone)	802.11 s/LoRa (backhaul)	---	Edge, fog, cloud	Rotary wing	Surveillance, agriculture, flight telemetry	Single UAV-to-Ground proof of concept
[62]	Single cluster	LoRa (access/backbone)	LoRa (backhaul)	---	Cloud	Rotary wing	Environmental emergencies	Single UAV two-hop proof of concept
[63]	Single cluster	LoRa (access/backbone)	LoRa (backhaul)	---	Cloud	Rotary wing	Open	Three UAVs two-hop proof of concept

**Table 7:** Proposed MAC protocols in FANETs involving LoRa or LoRaWAN.

Reference	Topology	Link Type	Communication Technology	Proposed MAC Protocol
[59,60]	Single cluster	Air-to-air	802.11 g (backhaul)	CSMA/CA
		Air-to-ground	LoRaWAN (access)	ALOHA
			802.11 g (backbone)	CSMA/CA
[61]	3D multi-layer	Air-to-air	802.11 s (backhaul)	CSMA/CA
			LoRa (backhaul)	TDMA
		Air-to-ground	802.11 s (as access/backbone)	CSMA/CA
			LoRaWAN (as access/backbone)	ALOHA
[62]	Single cluster	Air-to-air	LoRa (backhaul)	Custom slotted ALOHA
		Air-to-ground	LoRa (access/backbone)	Custom slotted ALOHA
[63]	Single cluster	Air-to-air	LoRa (backhaul)	CSMA/CA
		Air-to-ground	LoRa (access/backbone)	CSMA/CA

**Table 8:** Proposed routing protocols in FANETs involving LoRa or LoRaWAN.

Reference	Topology	Link Type	Communication Technology	Proposed Routing Protocol
[59,60]	Single cluster	Air-to-Air	802.11 g (backhaul)	OLSR
		Air-to-Ground	LoRaWAN (access)	---
			802.11 g (backbone)	OLSR
[61]	3D multi-layer	Air-to-Air	802.11 s (backhaul)	HWMP
			LoRa (backhaul)	Not defined
		Air-to-Ground	802.11 s (as access/backbone)	HWMP
			LoRaWAN (as access/backbone)	---
[62]	Single cluster	Air-to-Air	LoRa (backhaul)	GPS-based directed flooding
		Air-to-Ground	LoRa (access/backbone)	Not required
[63]	Single cluster	Air-to-Air	LoRa (backhaul)	Custom DSDV
		Air-to-Ground	LoRa (access/backbone)	Custom DSDV

**Table 9:** Energy consumption at different components of a FANET node during a 30-min flight.

Component	Current [mA]	Voltage [V]	Power [W]	Energy for a 30-min Flight [J]	% of Energy Consumption
Wi-Fi transmitter (802.11 n at 20 dBm, 2.4 GHz [89])	2000	5	10	18,000	4.83%
LoRa transmitter (RFM95 W at 20 dBm, 915 MHz [90])	120	3.7	0.32	799	0.21%
Communications controller (Raspberry Pi 4 B [91])	2500	5	12.5	22,500	6.04%
UAV (Parrot Anafi USA [92])	---	---	4 × 46	331,200	88.91%
TOTAL	---	---	125.1	372,499	100.00%

**Table 10:** Comparison between LoRa/LoRaWAN, Wi-Fi, and cellular LPWANs for their use in FANETs.

Aspect	LoRa/LoRaWAN	Wi-Fi	Cellular-Based LPWAN (NB-IoT)
Range [10,96]	2–5 km in urban areas and 15 km in suburban areas	Up to 100 m	0–1 km in urban areas and up to 15 km in suburban areas
Throughput [25,31,96]	Less than 200 kbps	Up to hundreds of Mbps	Up to 1 Mbps
Frequency bands	ISM (unlicensed)	ISM (unlicensed)	International Mobile Telecommunications (IMT) (licensed)
Energy consumption [J] *	~800	~18,000	~1500 [97]
Requires service provider infrastructure	No	No	Yes
Includes protocols for mesh networking	No	Yes	It does not require them as it relies on infrastructure

* Assuming parameters for maximum consumption during a 30-min period.

**Table 11:** Advantages and disadvantages of using LoRa in FANETs by aspect.

Aspect	Advantages	Disadvantages
Communications	Longer range Affordability Works on unlicensed frequency bands Open above physical layer	Lower data rate Lack of standardized upper-layer protocols suited for a mesh topology
Mobility [98]	Static Doppler shift immunity	It might require mitigation techniques at the physical layer for dynamic Doppler shift
Energy	Lower energy consumption	Energy consumption might be lower than necessary for higher data rate applications

**Table 12:** Advantages and disadvantages of using LoRa in FANETs by type of link.

Link	Advantages	Disadvantages
Air-to-air	Longer range Affordability Lower energy consumption	Lower data rate Lack of standardized upper-layer protocols suited for a mesh topology
Air-to-ground	Availability for IoT applications Lower energy consumption	Not as widespread as Wi-Fi or cellular for human-oriented communications

## Data Availability

Data is contained within the article.
